# Pre-therapy CT scan showing peritoneal thickening from metastatic renal pelvis carcinoma patients

**DOI:** 10.1007/s12032-018-1181-9

**Published:** 2018-08-16

**Authors:** Tzu-Yao Liao, Chuang-Chi Liaw, Ke-Hung Tsui, Yu-Hsiang Juan

**Affiliations:** 1 Division of Hemato-Oncology, Department of Internal Medicine, Chang-Gung Memorial Hospital, Chang-Gung University College of Medicine, 5, Fusing St., Gueishan Township, Taoyuan, 333 Taiwan, ROC; 2 Department of Urology, Chang-Gung Memorial Hospital, Chang-Gung University College of Medicine, Taoyuan, Taiwan, ROC; 3 Department of Medical Imaging and Intervention, Chang-Gung Memorial Hospital, Chang-Gung University College of Medicine, Taoyuan, Taiwan, ROC

**Keywords:** Renal pelvis cancer, Peritoneal thickening, Liver metastases, Abdominal wall invasion, Gastrointestinal complication, Computed tomography

## Abstract

We investigated clinical significance of peritoneal thickening from metastatic renal pelvis based on pretherapy computed tomography (CT) scan findings. The criteria for inclusion were as follows: (1) pathology and CT scan confirmed metastatic renal pelvis carcinoma and (2) peritoneal thickening based on pre-therapy CT scan findings. We investigated the route of spread, gastrointestinal (GI) complications, and response to chemotherapy. A total of 68 cases were enrolled in this study, including seven patients with liver metastases and three with abdominal wall invasion. GI complications included obstruction in ten patients and bleeding in three. Response to chemotherapy demonstrated by reduced peritoneal thickening was noted in 24 patients. In conclusion. peritoneal thickening with clinical suspicion of peritoneal involvement can get indirect evidence from route of spread (liver or abdominal wall), GI complications (obstruction or bleeding) or response to chemotherapy (obvious decrease peritoneal thickening) from metastatic renal pelvis carcinoma patients. Pretherapy CT scan with peritoneal thickening should be alert that tumor has spread to the peritoneum.

## Introduction

Approximately 20% of patients with upper urinary tract urothelial cancers reportedly have peritoneal metastases [1]. However, another report did not mention common sites of peritoneal metastases [2]. Precise diagnosis of peritoneal carcinomatosis is difficult based on imaging alone and histopathology is required for diagnosis [3, 4]. Parietal peritoneal thickening is an important characteristic of peritoneal carcinomatosis visible on computed tomography (CT) scans [3–7]. However, a wide variety of non-malignant etiologies, such as tuberculosis peritonitis, or a long period of peritoneal dialysis, can mimic peritoneal carcinomatosis [3, 4]. It is also difficult to quantify peritoneal thickening related to carcinomatosis using the tumor burden, such as in the response evaluation criteria for solid tumors (RECIST) [8]. The kidneys are a retroperitoneal organ located behind the parietal peritoneum, and renal pelvis urothelial cancers can spread anteriorly to the peritoneum from Gerota’s fascia (renal fascia) [9].

In the study, we retrospectively assess peritoneal thickening of metastatic renal pelvis carcinoma patients with clinical suspicion of peritoneal spread based on CT scan findings. We also investigated the route of spread, gastrointestinal (GI) complications, and chemotherapy response during the clinical course in these cases.

## Materials and methods

### Study population

**Fig. 1:**
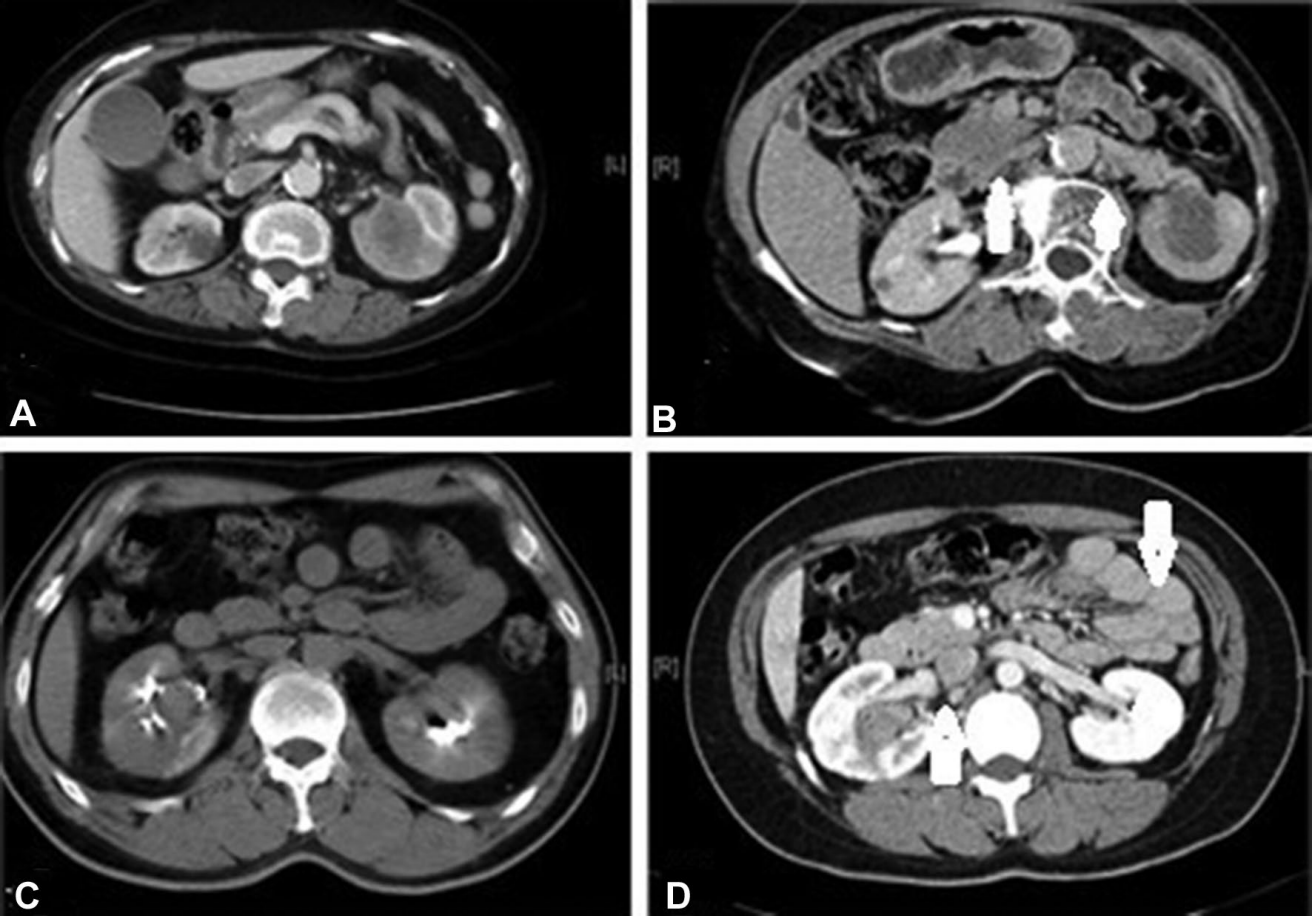
CT scans showing peritoneal thickening of renal pelvis carcinoma. Case 1: axial view of left renal pelvis malignancy showing **a** the absence of peritoneal thickening and **b** the presence of focal bowel wall thickening. Case 2: axial view of right renal pelvis malignancy showing **c** the absence of peritoneal thickening and **d** the presence of diffuse bowel wall thickening. Arrows indicate areas of peritoneal thickening

We conducted a retrospective case series study using data collected from patients with peritoneal thickening of metastatic renal pelvis cancer who were admitted to the oncology ward of Chang-Gung Memorial Hospital in Taoyuan, Taiwan, between January 2010 and December 2017. A single medical oncologist specializing in urological cancer provided most of the data. All patients were hospitalized due to chemotherapy treatment and admitted to palliative care for complications. CT scans were performed in all cases in order to evaluate the extent of the tumor. The criteria for inclusion included (1) pathology and CT scan confirmed metastatic renal pelvis carcinoma and (2) peritoneal thickening based on pre-therapy CT scan findings. Peritoneal thickening was defined as diffuse or focal bowel wall thickening. The CT scan findings showing peritoneal spread are presented in Fig. 1b, d.

### Evaluation items

We investigated patients with peritoneal thickening by evaluating the route of spread, GI complications, and response to chemotherapy. Route of spread included liver metastases and abdominal wall invasion. GI complications investigated were GI obstruction and GI bleeding. Response to chemotherapy was defined as an obvious decrease in peritoneal thickening following chemotherapy. Proximal GI obstruction was defined as obstruction level over the stomach or duodenum.

Chemotherapy regimens included 5-fluorouracil, leucovorin, cisplatin, and gemcitabine (Gemmis®; TTY, Taipei, Taiwan). When renal function impairment occurred (serum creatinine greater than 2 mg/dl), chemotherapy with continuous maintenance was administered with carboplatin replacing cisplatin.

## Results

A total of 71 consecutive patients with metastatic renal pelvis cancer collected in this study. Among them, 68 (94%) had peritoneal thickening; 38 men and 30 women aged between 39 and 88 years (median age, 65 years). Of them, 24 patients did not undergo nephroureterectomy procedures. Fifteen patients had multiple primary sites of urinary cancer, including five cases involving the bladder (one case simultaneously), nine cases involving the ureter (six cases simultaneously), and one case involving the bladder and the ureter. One case had received a previous renal transplantation, and one case had end-stage renal disease.

The patients’ clinical characteristics are shown in Table 1. The most common pathology in the study was urothelial carcinoma, and all patients exhibited various degrees of peritoneal thickening. Lung metastasis occurred in 44 patients and was the most common site of metastasis. Other metastatic sites included the liver (*n* = 7), bone metastases or direct lumbar spine invasion (*n* = 42), para-aortic lymph nodes (LNs; *n* = 36), left supraclavicular LNs (*n* = 5), the central nervous system (CNS; *n* = 3), and adrenal invasion (*n* = 3).

**Table 1:** Clinical characteristics of renal pelvis carcinoma patients with peritoneal thickening and suspicion of peritoneal spread (*n* = 68)

Characteristics	No. of patients (%)	
Age (years)		
Median (range)	65 (39–88)	
Sex		
Male/female	38/30	
Histology/cytology (*N* = 71)		
Urothelial carcinoma	64 (94)	
Squamous cell carcinoma	3 (4)	
Lympho-epithelial like carcinoma	1 (1)	
Tumor spread		
Lung	44	
Liver	7	
Bone/direct lumbar vertebra body invasion	42	
Supraclavicular LNs	5	
CNS	3	
Adrenal gland invasion	3	
Para-aortic LN involvement	36	
Suspect peritoneal spread	Total	Initial
Related to liver metastasis	7	4
Related to abdominal wall invasion	3	1
Related to GI obstruction	10	3
Related to upper GI bleeding	3	0
Response to systemic chemotherapy	24	

*LN* lymph node, *CNS* central nervous system, *GI* gastrointestinal

**Fig. 2:**
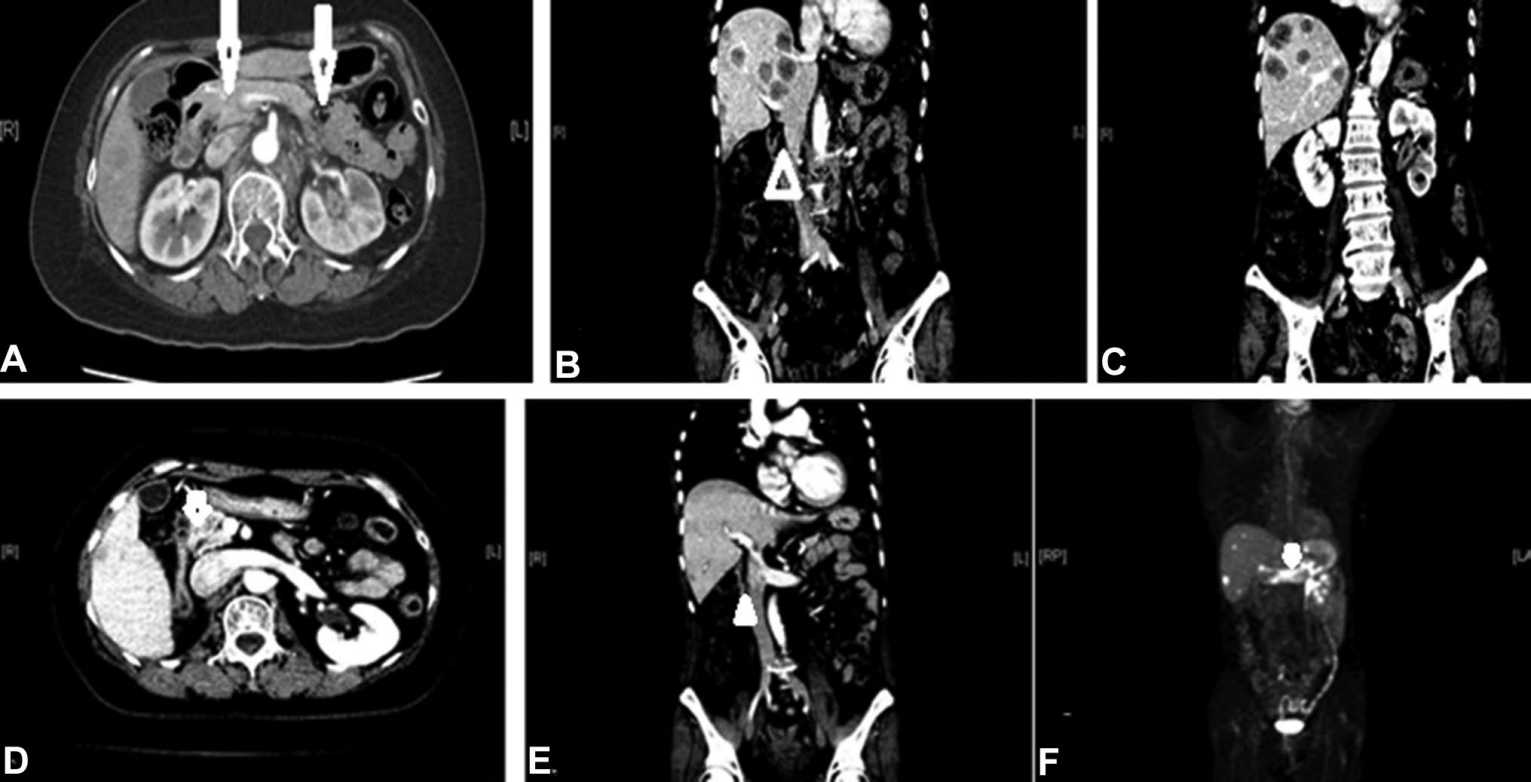
CT scans showing peritoneal thickening of renal pelvis carcinoma. Case 1: initial presentation with liver metastases; **a** axial and **b, c** coronal views showing peritoneal thickening anextra-portal vein lesion with multiple liver metastases. Case 2: relapse with liver metastases; **d** axial and **e** coronal views showing peritoneal thickening with liver metastases. **f** PET scan showing peritoneal lesion with multiple liver metastases. Arrows indicate thickening of the peritoneum. Arrowheads indicate extra-portal vein lesions

**Fig. 3:**
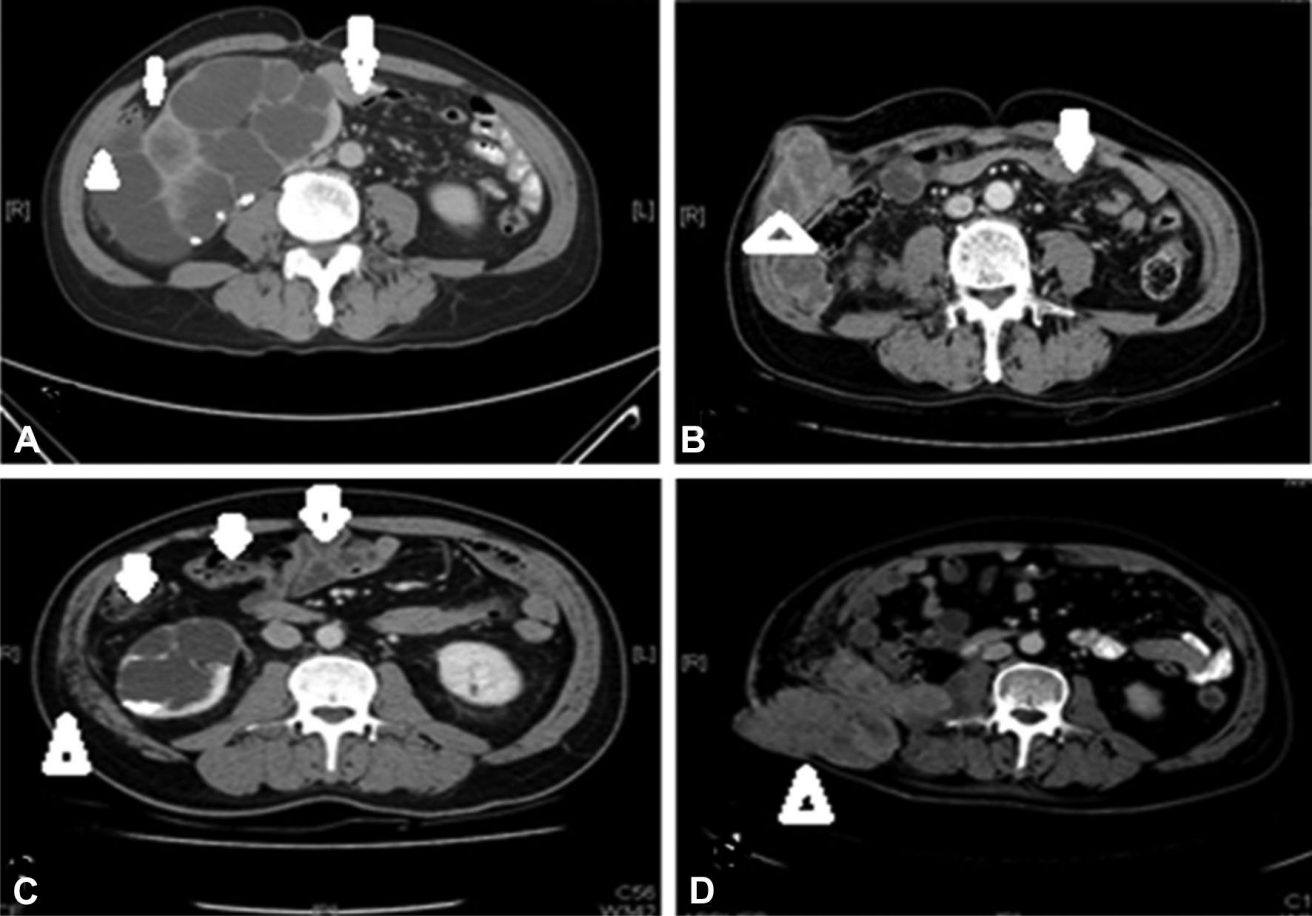
CT scans showing peritoneal thickening of renal pelvis carcinoma. Axial views of Case 1 (**a, b**) and Case 2 (**c, d**) before nephroureterectomy, all showing peritoneal thickening with lateral abdominal wall invasion. **b** Axial view showing tumor progression with an obvious right lateral abdominal wall mass following surgery and chemotherapy. Arrows indicate a thickened or dirty peritoneum. Arrowheads indicate abdominal wall invasion lesions

Liver metastases were noted in seven patients with clinical suspicion of peritoneal spread, including four patients showing an initial presentation. Figure 2a–c presents one case with an initial presentation of liver metastases, and Fig. 2d–f reveals another case where liver metastases developed later. Both patients had extra-portal lesions related to peritoneal involvement. Furthermore, the three patients with abdominal wall invasion were all detected initially. Two cases (Fig. 3a–d, respectively) showed lesions with abdominal wall invasion due to peritoneal involvement.

**Fig. 4:**
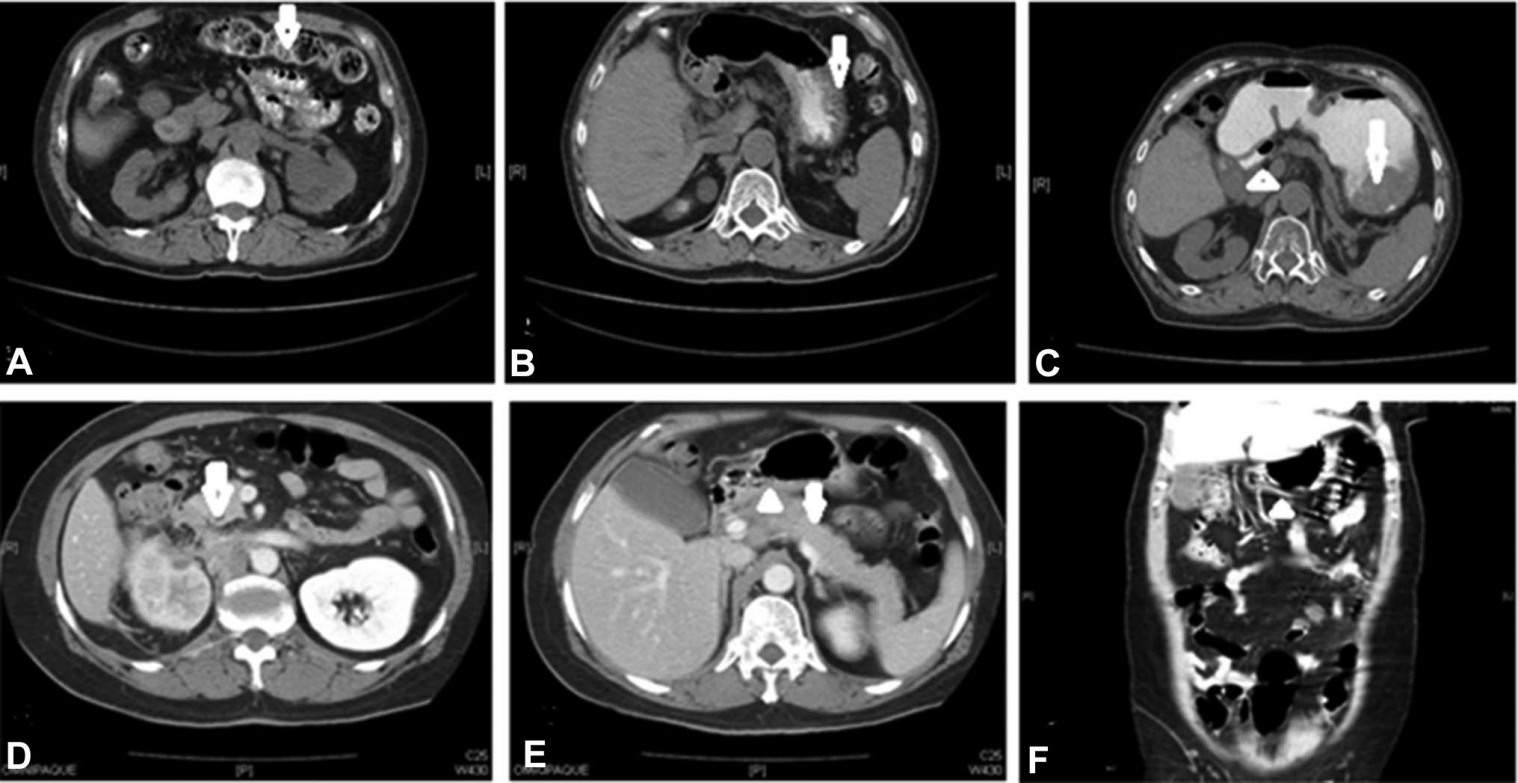
CT scans showing peritoneal thickening of renal pelvis carcinoma complicated by gastrointestinal obstruction. Axial views of Case 1 (**a, b**) and Case 2 (**c**–**e**) before nephroureterectomy showing a thickened or dirty peritoneum with stomach wall infiltration. **c** Axial view showing tumor progression with obvious stomach wall infiltration and proximal gastrointestinal obstruction. **d, e** Axial view and **f** coronal view showing thickening of the peritoneum with initial presentation of proximal gastrointestinal obstruction. Arrows indicate a thickened or dirty peritoneum. Arrowheads indicate tumors with gastrointestinal tract obstruction

Regarding GI complications, ten patients had obstructions and three had bleeding. GI obstruction included patients with proximal GI obstruction (*n* = 8), initial presentations (*n* = 3), those who received surgery (*n* = 2), those who received bypass surgery (*n* = 1), and those who received ileostomy (*n* = 1). Figure 4a–c shows one case showing peritoneal spread with gastric wall involvement that later developed into proximal GI obstruction. Figure 4d–f presents another case with an initial presentation of peritoneal spread with proximal GI obstruction who then received bypass surgery.

**Fig. 5:**
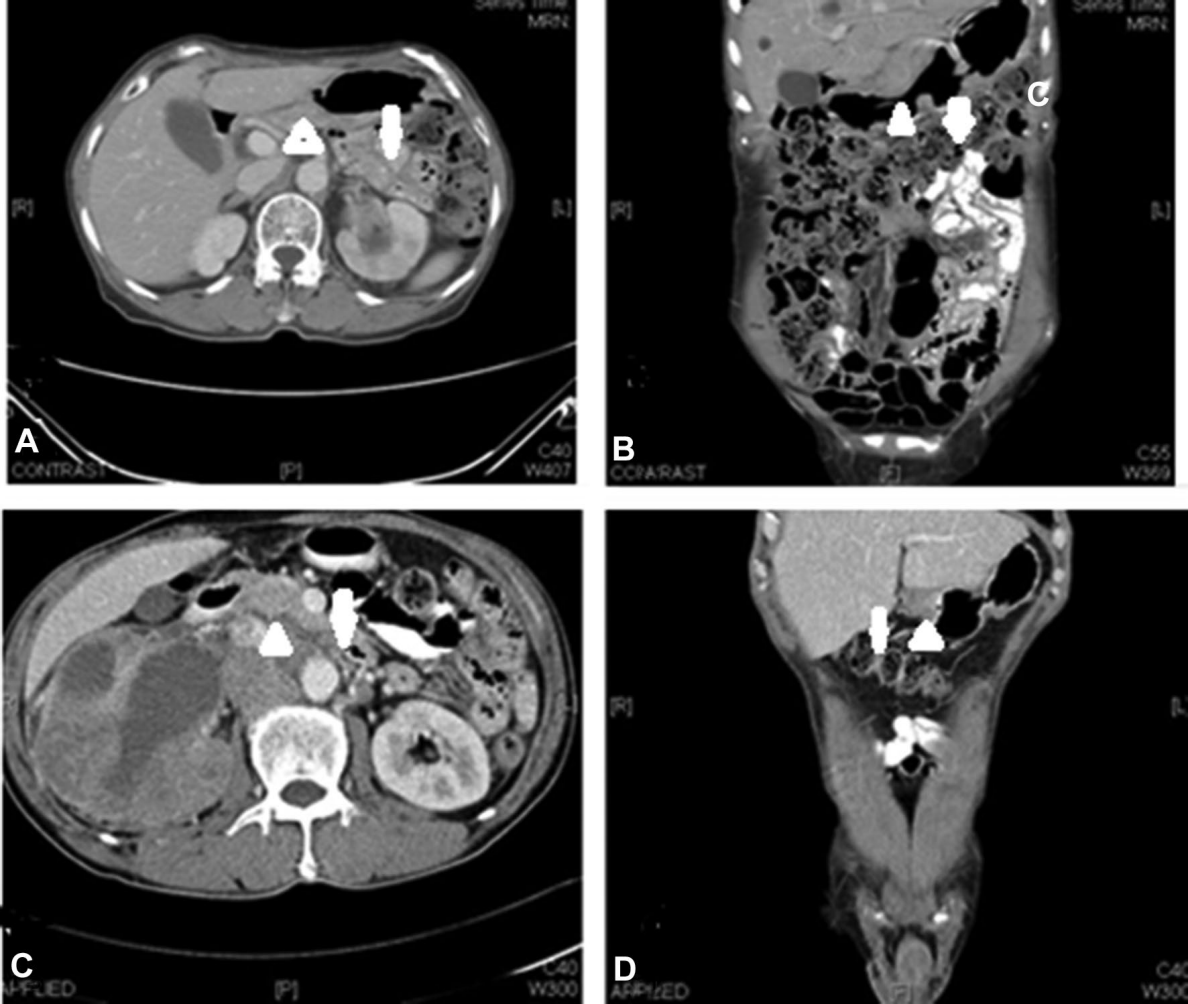
CT scans showing peritoneal thickening of renal pelvis carcinoma complicated by gastrointestinal bleeding. Case 1: **a** axial and **b** coronal views. Case 2: **c** axial and **d** coronal views. **a**–**d** All show thickened peritoneum and tumor invasion into gastrointestinal tract. Arrows indicate a thickened or dirty peritoneum. Arrowheads indicate tumor invasion into the gastrointestinal tract

Of the three patients with GI bleeding, all of which were of upper GI origin, one had an initial presentation. Two cases (Fig. 5a–d, respectively) showed peritoneal spread with invasion into the duodenum.

**Fig. 6:**
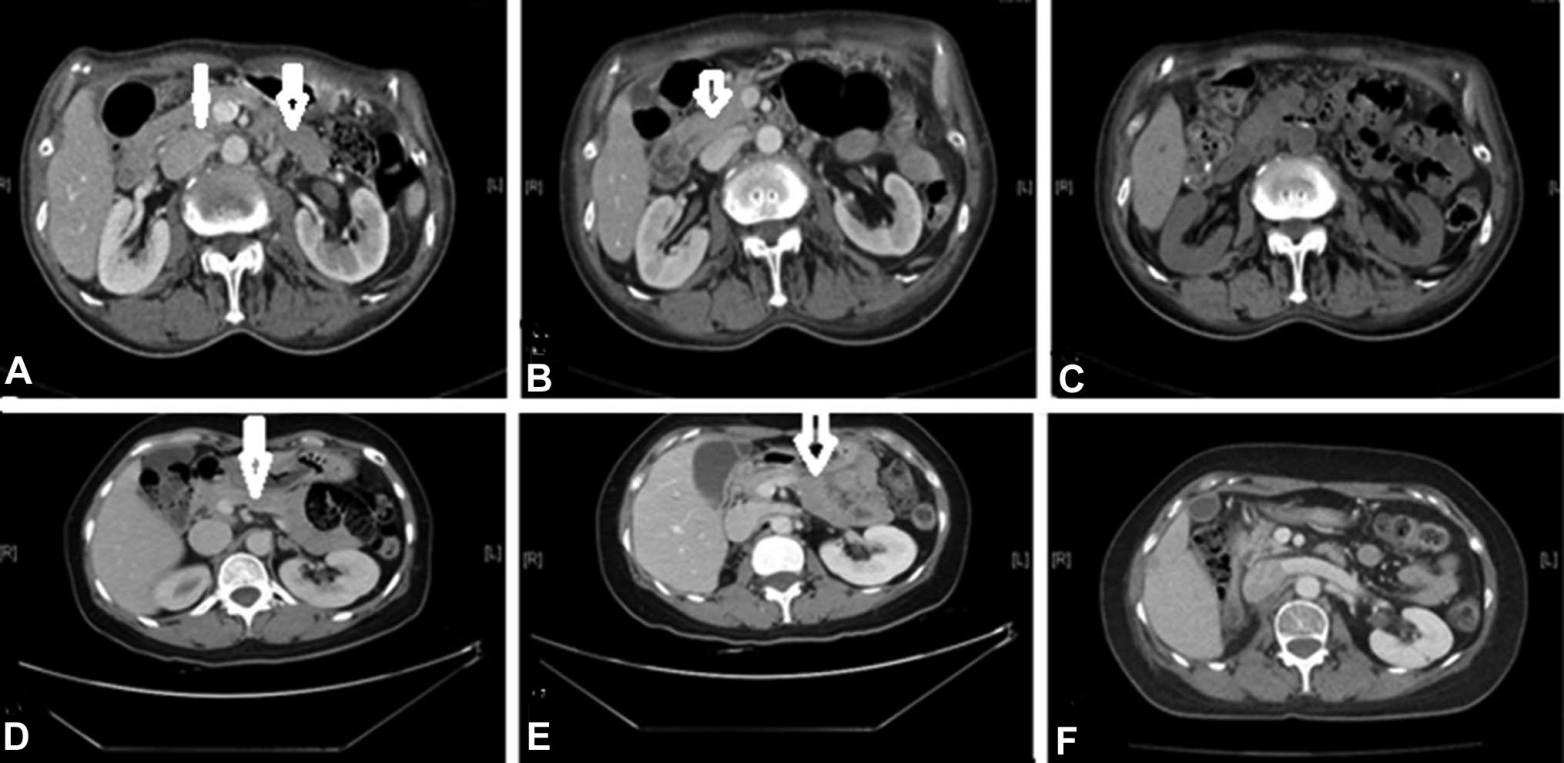
CT scans showing peritoneal thickening of renal pelvis carcinoma. Case 1: axial views **a** 12 months before chemotherapy, showing thickened peritoneum; **b** 12 months after chemotherapy, showing decreased peritoneal thickening; and **c** 21 months after chemotherapy, showing an obvious reduction in peritoneal thickening. Case 2: axial views **d** before nephroureterectomy, showing thickened peritoneum; **e** after nephroureterectomy, showing increased peritoneal thickening; and **f** three years after nephroureterectomy and chemotherapy, showing obviously reduced peritoneal thickening. Arrows indicate thickened peritoneum

Response to chemotherapy was noted in peritoneal lesions of 24 patients. Two cases (Fig. 6a–f, respectively) showed an obvious decrease in peritoneal thickening following chemotherapy.

## Discussion

Based on CT scan findings from 71 consecutive patients with metastatic renal pelvis cancer, 94% had peritoneal thickening with clinical suspicion of peritoneal spread of renal pelvis carcinoma. The discrepancies between our study and existing literature probably relate to the lack of histological diagnosis and relying on detection of peritoneal metastases from obvious image findings or presentation of clinical symptoms. Peritoneal involvement of renal pelvis carcinoma is most likely related to direct spread from Gerota’s fascia (renal fascia) [4–7].

In our study, the clinical evidence for peritoneal thickening in renal pelvis carcinoma patients with suspicion of peritoneal spread was got as indirect evidence from the route of spread, GI complications, and response to chemotherapy. In colorectal tumors, which predominantly spread along the mesenteric circulation to the liver, cancer cells enter the liver via the portal vein and likely give rise to liver metastases [10, 11]. Similarly, our patients had extra-portal lesions related to peritoneal involvement and exhibited development of liver metastases [10, 11]. Similar to bladder urothelial carcinoma abdominal wall invasion related to adjacent peritoneal seedings [12]. Three of our patients initially demonstrated abdominal wall invasion with a thickened or dirty peritoneum.

Bowel obstructions are often observed in patients with peritoneal carcinomatosis from metastatic carcinoma [13]. GI obstruction occurred in patients with a thickened or dirty peritoneum and clinical suspicion of peritoneal spread. Furthermore, proximal GI obstructions were predominant, and some cases required surgical intervention.

Upper GI bleeding due to renal pelvis carcinoma invasion of the duodenum or stomach has been reported in the literature [14, 15]. Similarly, tumor invasion into the duodenum was detected in the patients with a thickened peritoneum and clinical suspicion of peritoneal spread.

The tumor burden in patients with peritoneal carcinomatosis is difficult to quantify, as the response in patients with a thickened peritoneum and clinical suspicion of peritoneal spread is based on image findings. An obvious reduction of the thickened peritoneum following chemotherapy may represent tumor response. In this study, 24 patients showed an obvious decrease in peritoneal thickening following chemotherapy.

Because the diagnosis of peritoneal spread merely based on image findings due to the lack of a pathological diagnosis. These patients resemble uremia patients with long-term peritoneal dialysis that can lead to fibrotic thickening of the peritoneal membrane [3, 4]. Peritoneal fibrosis is associated with the appearance of myofibroblasts and extracellular matrix expansion [16]. Quantitative analysis of the inhibitory effects of tumor cells can be determined from human fibroblasts [17], whereby cancer-associated fibroblasts (CAFs) have been shown to also have a prominent role in the progression, growth, and spread of cancers [18]. While CAFs are linked to cytokine production by inflammatory cells [18]. The clinical significance of peritoneal thickening remains unclear. We hypothesized that peritoneal thickening could be a protective effect to inhibit tumor growth. However, cancer cells hidden by peritoneal thickening promote tumor progression upon initiation of the inflammatory process.

There were several limitations to our study. First, case series data were collected retrospectively. Second, the peritoneal thickening diagnoses were based only on CT scans and were not confirmed by pathology.

## Conclusion

Peritoneal thickening with clinical suspicion of peritoneal involvement can get indirect evidence from route of spread (liver or abdominal wall), GI complications (obstruction or bleeding) or response to chemotherapy (obvious decrease peritoneal thickening) from metastatic renal pelvis carcinoma patients. Pretherapeutic CT scan with peritoneal thickening should be alert that tumor has spread to the peritoneum.
